# Influenza vaccination: opinions of health care professionals working in pediatric emergency departments

**DOI:** 10.1186/s13052-019-0638-6

**Published:** 2019-04-11

**Authors:** Luciano Pinto, Raffaele Falsaperla, Alberto Villani, Giovanni Corsello, Roberto Del Gado, Adolfo Mazzeo, Riccardo Lubrano

**Affiliations:** 1 Società Italiana di Medicina Emergenza Urgenza Pediatrica, Via Nevio 60, 80122 Napoli, Italy; 2 Policlinico-Vittorio Emanuele Università di Catania, UOC di Pediatria e Neonatologia, Catania, Italy; 3 Ospedale Pediatrico Bambino Gesù, UOC di Pediatria Generale e Malattie Infettive, Roma, Italy; 4 Università degli Studi di Palermo, Clinica Pediatrica Palermo, Palermo, Italy; 5 Università degli Studi della Campania “Luigi Vanvitelli”, Caserta, Italy; 6 Università degli Studi di Roma “La Sapienza” UOC di Pediatria e Neonatologia, Polo di Latina, Roma, Italy

**Keywords:** Influenza vaccination, Health care professionals, Vaccine coverage, Italy

## Abstract

**Background:**

Vaccine coverage of health care professionals against influenza is still low in Italy, as well as in other European countries.

**Methods:**

Between March and May 2018, this study was performed to collect the opinions of Pediatric health care professionals, working in emergency departments, regarding the efficacy and safety of the influenza vaccine. An anonymous online survey was employed to evaluate socio-demographic and professional characteristics, knowledges, beliefs and attitudes.

**Results:**

Five hundred four health care professionals completed the survey: 331 physicians, 140 nurses and 33 other health are professionals. During the 2017–18 season, 55.8% of physicians, 19.3% of nurses and 12.1% of other health care professionals had vaccinated against the influenza virus. Not vaccinated physicians and nurses with less than 40 years of age were fewer than not vaccinated physicians and nurses with more than 40 years of age. Nurses and other health care professionals were less trustworthy of the influenza vaccination, less aware of the possibility of contracting and transmitting influenza and other vaccine-preventable diseases.

**Conclusions:**

Insufficient adherence to the influenza vaccination in physicians, nurses and other health care professionals is a concern for those assisting high-risk patients, especially in emergency departments. Therefore, it is vital to promote education of health care professionals and students regarding vaccinations. High vaccine coverage should be embedded in the safe hospital paradigm and should become a goal for the hospital's directors.

## Background

Influenza vaccination of health care professionals is the most effective public health strategy to prevent influenza’s transmission in hospital [1], reduce the mortality of elderly and high-risk patients [2] and limit absences from work during influenza epidemics [3–5].

Health care professionals, by being in contact with potentially infected patients or materials, can contract influenza and spread the virus to their patients, their families and susceptible colleagues. Health care professionals are at higher risk of contracting influenza compared to healthy adults not working in health care contexts [6]. During each season, 20% of health care professionals are estimated to contract influenza [7], often continuing working although infected [8], hence favoring the spread of the virus.

The majority of countries recommends annual influenza vaccination for health care professionals but a large number of professionals do not vaccinate. In European countries vaccine coverage is still low (between 5 and 54.9%, with a median of 25.7% in 2014–15) [9]. In the United States 78.4% of health care personnel reported having received an influenza vaccination during the 2017–18 season, but vaccination coverage was highest (94.8%) among health care personnel working in settings where vaccination was required [10].

In Italy data on vaccine coverage of health care professionals is quite limited. In the region Veneto there has been a moderate increase in the last years (from 16.7% in 2013–14 to 28.8% in 2017–18) [11], and several studies have shown equivalent variations also in other regions [12]. Nevertheless, vaccine coverage for influenza is clearly far from the 75% target established by the European Commission for high-risk groups [12–17].

Such matter is relevant especially in pediatric emergency departments, general pediatric wards and intensive care units. Indeed, since the 2009 H1N1 influenza pandemic, it is known that health care professionals, especially physicians, have a higher risk of contracting influenza in these contexts [18].

During seasonal influenza epidemics, air and surfaces of emergency departments are contaminated with the influenza virus [19] and the possibility of being infected in the emergency department is 3.4 higher (OR 3.4; IC 95%, 1.27–9.1) than in the operating room [20].

These considerations encouraged the Italian Society of Pediatric Emergency Medicine (SIMEUP) and the Italian Society of Pediatrics (SIP), to publicize an online survey among Pediatric health care professionals working in the emergency department in order to collect their opinion on vaccines’ efficacy and safety, with a specific focus on influenza vaccination.

## Methods

An anonymous online survey composed of 4 sections was developed. In the first section, socio-demographic and professional characteristics of the participants were asked: age range, sex, professional role, department and region of work. In the second section, vaccination status was asked, with specific questions on measles, rubella, mumps, varicella, hepatitis B, influenza, meningococcus (B, C, C-A-Y-W) and pneumococcus. In the third section, participants were asked to agree on statements concerning influenza, influenza vaccination and, more widely, vaccinations. In the fourth section, the following items were asked: the existence of training activities on vaccination, the knowledge on the existence of quarantine measures in their hospital for susceptible Health care professionals, their opinion on mandatory vaccinations for Health care professionals and, at the end, an optional evaluation of the survey.

The survey was elaborated on Google Forms and was circulated on the SIMEUP’s website to all health care professionals (both members and non-members of the society). The survey was also sent by email to all members of SIMEUP and on the newsletter of SIMEUP, in the period between March and May 2018.

Results were analyzed with descriptive statistics, using absolute frequency with percentages for categorical variables and mean with standard deviation (S.D.) for continuous variables.

Attitudes and beliefs were analyzed with a five-point Likert scale, ruling out participants who had not expressed their opinion and grouping the other participants in two categories, “Strongly disagreeing and Disagreeing” and “Strongly agreeing and Agreeing”. Odds Ratio (O.R.) was measured between nurse and physicians.

The Parent Attitudes about Childhood Vaccines (PACV) Short Scale [21, 22] was used to evaluate Vaccine Hesitancy in health care professionals.

Statistical analysis was performed using MedCalc Statistical Software version 16.4.3 (MedCalc Software bvba, Ostend, Belgium; https://www.medcalc.org; 2016).

## Results

Five hundred four Health care professionals completed the survey. Three hundred thirty-one were physicians (among them 103 were residents), 140 were nurses and 33 were health care professionals with other roles (‘Other’). Participants worked in disparate geographical areas of Italy (Table 1).

**Table 1:** Socio-demographic characteristics of the participants

		Physicians	Nurses	Other Health Care professionals	Total
Number of answers		331	140	33	504
Sex	Males	122	37	11	170 (33.7%)
	Females	209	103	22	334 (66.3%)
Age range	20–29	67	54	14	135 (27.2)
	30–39	105	27	5	137 (15.1%)
	40–49	32	36	8	76 (15.1%)
	50–59	63	21	5	89 (17.7%)
	60–69	58	2	1	61 (12.1%)
	70–79	6			6 (1.2%)
Geographic area	North	134	47	9	190 (37.7%)
	Center	79	34	3	116 (23.0%)
	South and Islands	118	59	21	198 (39.3%)

55.8% of physicians (185/331), 19.3% of nurses (27/140), and 12.1% of health care professionals with other roles (4/33) had vaccinated against influenza in 2017–18 (Table 2).

**Table 2:** Vaccinated Health Care professionals (number of vaccinated/total and % of vaccinated) in 2017/18 divided by role and geographic area

Role	North	Center	South and Islands	Total
Others	2/9 (22.2%)	1/3 (33.3%)	1/21 (4.8%)	33 (12.1%)
Nurses	5/47 (10.6%)	13/34 (38.2%)	9/59 (15.3%)	140 (19.3%)
Physicians	79/134 (58.9%)	52/79 (65.8%)	54/118 (45.8)	331 (55.9%)
Total	86/190 (45.3%)	66/116 (56.9%)	64/198 (32.3%)	504 (42.8%)

**Fig. 1:**
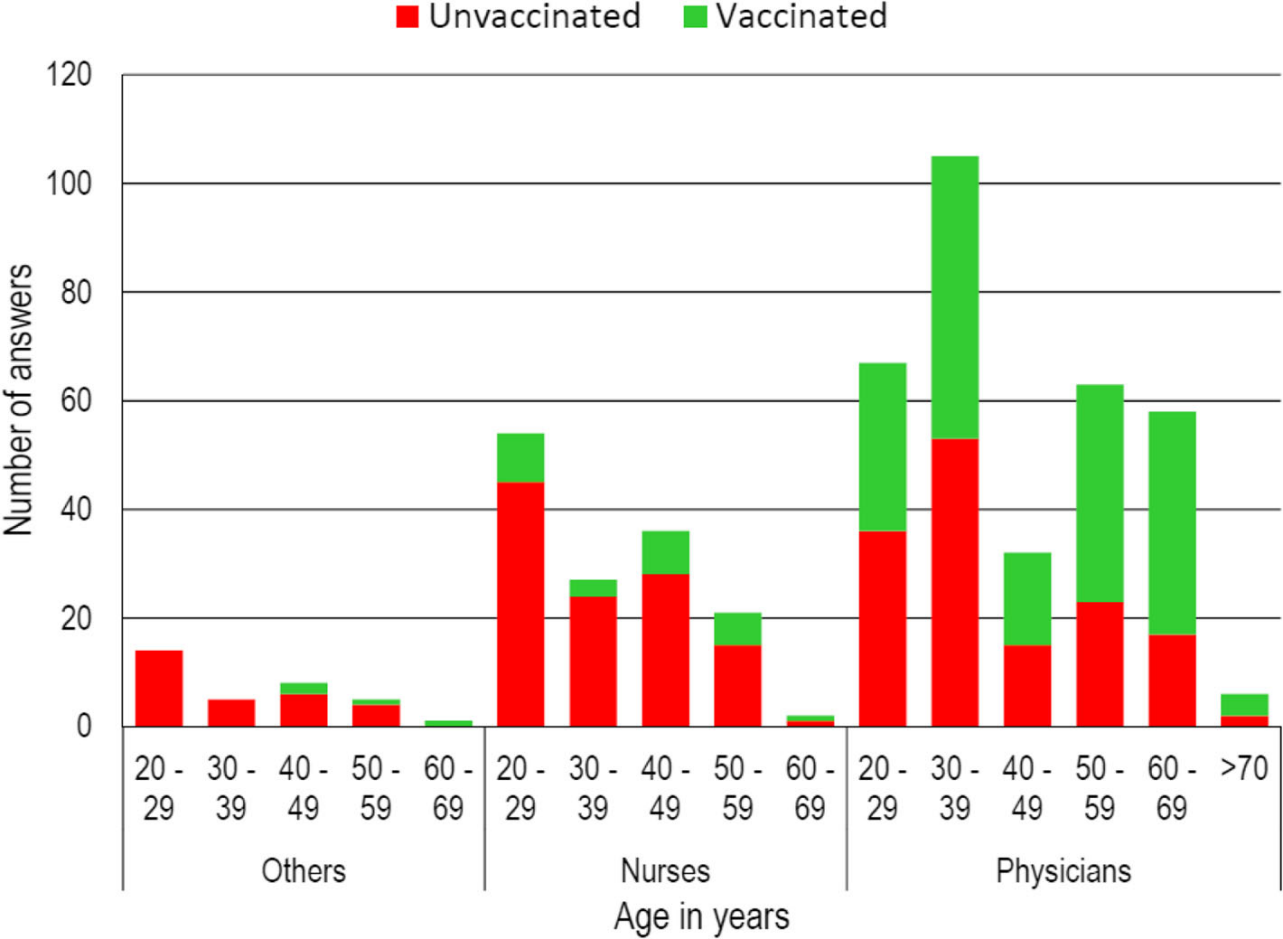
Health Care professionals vaccinated for influenza in 2017–2018. Distribution by role and age range

Not vaccinated physicians and nurses with less than 40 years of age were fewer than not vaccinated physicians and nurses with more than 40 years of age (Table 3, Fig. 1).

**Table 3:** Adherence to the influenza vaccination in Health Care professionals according to age in 2017/18

Physicians				Nurses			
Age in years	Vaccinated			Age in years	Vaccinated		
	No	Yes			No	Yes	
20–39	89 (51.7%)	83 (48,3%)	O.R. 0.52 I.C. 95% 0.34 – 0.81 P = 0.0038	20–39	69 (85.2%)	12 (14.8%)	O.R. 0.51 I.C. 95% 0.21–1.19
> 40	57 (35.8%)	102 (64.2%)		> 40	44 (74.6%)	15 (25.4%)

28.6% of nurses, 11.5% of physicians and 27.3% of other health care professionals deemed the risk of contracting influenza as low (nurses and physicians, O.R. 3,08; IC 95% 2,05-6,47; *p* < 0.0001) whereas 22.1% of nurses, 7.3% of physicians and 27.3% of other health care professionals deemed the risk of transmitting influenza as low (between physicians and nurses O.R. 3,64; IC 95% 2,05-6,47; *p* < 0.0001).

90.9% of physicians, 75,7% of nurses and 72.7% of other health care professionals were not afraid about the influenza vaccination causing ‘severe damages’. 26.3% of physicians and 36.4% of nurses felt vaccine information to be insufficient. 66.2% of physicians considered appropriate for the influenza vaccination to be required to work as health care professionals, compared to 42.9% of nurses (O.R. 0.33, IC 85% 0.21–0.53) and 36.4% of other health care professionals (Tables 4 and 5).

**Table 4:** Levels of agreement on statements regarding the influenza vaccine

Statements regarding the influenza vaccine	Role	Not agreeing (1 + 2)		Uncertain		Agreeing (4 + 5)		Total	Mean + Standard Deviation (IC 95%)
		N°	%	N°	%	N°	%		
I believe that my risk of contracting influenza is low	Others	15	45.5	9	27.3	9	27.3	33	2.64 + 1.39 (2.14–3.13)
	Nurses	64	45.7	36	25.7	40	28.6	140	2.75 + 1.29 (2.53–2.96)
	Physician	273	82.5	20	6.0	38	11.5	331	1.73 + 1.13 (1.61–1.86)
	Total	352	69.8	65	12.9	87	17.3	504	2.08 + 1.28 (1.96–2.19)
I believe that the risk of transmitting influenza to one of my patients is low	Others	16	48.5	8	24.2	9	27.3	33	2.67 + 1.41 (2.17–3.17)
	Nurses	81	57.9	28	20.0	31	22.1	140	2.45 + 1.27 (2.24–2.66)
	Physicians	287	86.7	20	6.0	24	7.3	331	1.64 + 0.99 (1.53–1.74)
	Total	384	76.2	56	11.1	64	12.7	504	1.93 + 1.17 (1.83–2.03)
I am afraid of ‘severe damages’ caused by the influenza vaccination	Others	24	72.7	7	21.2	2	6.1	33	1.79 + 0.99 (1.44–2.14)
	Nurses	106	75.7	16	11.4	18	12.9	140	1.91 + 1.22 (1.70–2.11)
	Physicians	301	90.9	9	2.7	21	6.3	331	1.43 + 0.96 (1.27–1.59)
	Total	431	85.5	32	6.3	41	8.1	504	1.59 + 1.06 (1.49–1.68)
Information on the influenza vaccine is limited	Other	17	51.5	9	27.3	7	21.2	33	2.48 + 1.15 (2.07–2.89)
	Nurses	57	40.7	32	22.9	51	36.4	140	2.65 + 1.36 (2.70–3.16)
	Physicians	188	56.8	56	16.9	87	26.3	331	2.43 + 1.3 (2.21–2.57)
	Total	262	52.0	97	19.2	145	28.8	504	2.57 + 1.32 (2.46–2.69)
I consider appropriate for the influenza vaccine to be required to work as Health care professionals	Others	16	48.5	5	15.2	12	36.4	33	2.97 + 1.51 (2.48–3.46)
	Nurses	48	34.3	32	22.9	60	42.9	140	3.11 + 1.46 (2.87–3.35)
	Physicians	58	17.5	54	16.3	219	66.2	331	3.85 + 1.32 (3.63–4.07)
	Total	122	24.2	91	18.1	291	57.7	504	3.59 + 1.42 (3.46–3.71)

**Table 5:** Levels of agreement on statements regarding the influenza vaccine between nurses and physicians

Statements regarding the influenza vaccine	Role	Agreeing				
		N°	%	OR	I.C. 95%	p
I believe that my risk of contracting influenza is low	Nurses	40	28.57	3.08	1.87–5.08	*P* < 0.0001
	Physician	38	11.48			
I believe that the risk of transmitting influenza to one of my patients is low	Nurses	31	22.14	3.64	2.05–6.47	*P* < 0.0001
	Physicians	24	7.25			
I am afraid of ‘severe damages’ caused by the influenza vaccination	Nurses	18	12.86	0.25	0.15–041	*P* = 0.0215
	Physicians	21	6.34			
Information on the influenza vaccine is limited	Nurses	51	36.43	1.93	1.23–3.05	*P* = 0.0045
	Physicians	87	26.28			
I consider appropriate for the influenza vaccine to be required to work as Health care professionals	Nurses	60	42.86	0.33	0.21–0.53	*P* = 0.0001
	Physicians	219	66.16			

37.9% of nurses and 14.2% of physicians deemed the risk of contracting a vaccine-preventable disease as low (O.R. 4.26, IC 95% 2.63–6.89; *p* < 0.0001). According to 30.7% of nurses and 13.0% of physicians (O.R. 4.03; IC 95% 2.44–6.66; *p* < 0.0001), the risk of transmitting a vaccine-preventable disease was low. 58.6% of nurses, 66.7% of other health care professionals and 89.1% of physicians were not afraid of side effects caused by vaccines. 19.3% of nurses and 7.5% of physicians did not trust information on vaccines. The majority of nurses (64.3%) and physicians (76.7%) deemed appropriate for vaccinations to be a required to work as health care professionals; 33.3% of nurses deemed the number of vaccines administered during one visit as excessive (physicians: 9.4%) and 21.4% of nurses preferred natural active immunity to acquired active immunity (physicians: 4.5%). 87.3% of physicians had no doubts about vaccinations, compared to 54.3% of nurses and 54.5% of other health care professionals (Tables 6 and 7).

**Table 6:** Levels of agreement on statements regarding vaccines

Statements regarding vaccines	Role	Not agreeing (1 + 2)		Uncertain		Agreeing (4 + 5)		Total	Mean +/- Standard Deviation (IC 95%)
		N°	%	N°	%	N°	%		
I believe that my risk of contracting a vaccine-preventable disease is low	Others	17	51.52	11	33.33	5	15.15	33	2.48 + 1.20 (2.06–2.91)
	Nurses	64	45.71	23	16.43	53	37.86	140	2.88 + 1.41 (2.64–3.11)
	Physicians	262	79.15	22	6.65	47	14.20	331	1.89 + 1.20 (1.76–2.02)
	Total	343	68.06	56	11.11	105	20.83	504	2.20 + 1.33 (2.09–2.32)
I believe that the risk of transmitting a vaccine-preventable disease to one of my patients is low	Others	12	36.36	9	27.27	12	36.36	33	2.97 + 1.33 (2.50–3.44)
	Nurses	65	46.43	32	22.86	43	30.71	140	2.73 + 1.30 (2.51–2.95)
	Physicians	262	79.15	26	7.85	43	12.99	331	1.85 + 1.14 (1.73–1.98)
	Total	339	67.26	67	13.29	98	19.44	504	2.17 + 1.28 (2.06–2.28)
Vaccines’ benefits are uncertain	Others	19	57.58	4	12.12	10	30.30	33	2.64 + 1.54 (2.09–3.18)
	Nurses	89	63.57	20	14.29	31	22.14	140	2.31 + 1.31 (2.09–2.53)
	Physicians	293	88.52	8	2.42	30	9.06	331	1.52 + 1.10 (1.40–1.64)
	Total	401	79.56	32	6.35	71	14.09	504	1.81 + 1.28 (1.70–1.92)
Vaccines should be required to work as Health Care professionals	Others	5	15.15	3	9.09	25	75.76	33	3.94 + 1.20 (3.51–4.36)
	Nurses	24	17.14	26	18.57	90	64.29	140	3.78 + 1.31 (3.56–4.00)
	Physicians	35	10.57	42	12.69	254	76.74	331	4.11 + 1.13 (3.99–4.23)
	Total	64	12.70	71	14.09	369	73.21	504	4.01 + 1.19 (3.90–4.11)
I am afraid of side effects caused by vaccinations	Others	22	66.67	4	12.12	7	21.21	33	2.21 + 1.36 (1.73–2.69)
	Nurses	82	58.57	26	18.57	32	22.86	140	2.35 + 1.36 (2.12–2.58)
	Physicians	295	89.12	16	4.83	20	6.04	331	1.54 + 0.93 (1.41–1.66)
	Total	399	79.17	46	9.13	59	11.71	504	1.81 + 1.16 (1.71–1.91)
** I trust the information I received on vaccines	Others	8	24.24	4	12.12	21	63.64	33	3.61 + 1.32 (3.14–4.07)
	Nurses	27	19.29	28	20.00	85	60.71	140	3.61 + 1.21 (3.82–4.24)
	Physicians	25	7.55	25	7.55	281	84.89	331	4.24 + 1.05 (4.13–4.35)
	Total	60	11.90	57	11.31	387	76.79	504	4.02 + 1.15 (3.92–4.13)
** Becoming immune naturally by contracting a disease is better than becoming immune with vaccinations	Others	18	54.55	6	18.18	9	27.27	33	2.61 + 1 .34 (2.13–3.08)
	Nurses	84	60.00	26	18.57	30	21.43	140	2.31 + 1.35 (2.09–2.54)
	Physicians	297	89.73	19	5.74	15	4.53	331	1.49 + 0.89 (1.39–1.59)
	Total	399	79.17	51	10.12	54	10.71	504	1.79 + 1.14 (1.69–1.89)
** Children should receive less vaccines in one visit	Others	13	39.39	9	27.27	11	33.33	33	2.88 + 1.36 (2.40–3.36)
	Nurses	61	43.57	32	22.86	47	33.57	140	2.80 + 1.39 (2.57–3.03)
	Physicians	259	78.25	41	12.39	31	9.37	331	1.78 + 1.13 (1.66–1.90)
	Total	333	66.07	82	16.27	89	17.66	504	2.14 + 1.32 (2.02–2.25)
** Children receive too many vaccinations	Other	21	63.64	4	12.12	8	24.24	33	2.33 + 1.22 (1.90–2.77)
	Nurses	94	67.14	25	17.86	21	15.00	140	2.11 + 1.24 (1.91–2.32)
	Physicians	304	91.84	13	3.93	14	4.23	331	1.42 + 0.85 (1.33–1.51)
	Total	419	83.13	42	8.33	43	8.53	504	1.67 + 1.06 (1.58–1.75)
** Overall, do you have doubts/worries regarding vaccines? 1 (a lot of doubts) to 5 (no doubts at all)	Others	11	33.33	4	12.12	18	54.55	33	3.30 + 1.29 (2.85–3.76)
	Nurses	40	28.57	24	17.14	76	54.29	140	3.37 + 1.24 (3.16–3.58)
	Physicians	29	8.76	13	3.93	289	87.31	331	4.25 + 0 .99 (4.14–4.35)
	Total	80	15.87	41	8.13	383	75.99	504	3.94 + 1.17 (3.84–4.04)

**Statements used to elaborate the PACV Short Scale

**Table 7:** Levels of agreement on statements regarding vaccines between nurses and physicians

Statements regarding vaccines	Role	Agree or Strongly Agree				
		N°	%	OR	I.C. 95%	p
I believe that my risk of contracting a vaccine-preventable disease is low	Nurses	53	37.86	4.26	2.63–6.89	*P* < 0.0001
	Physicians	47	14.20			
I believe that the risk of transmitting a vaccine-preventable disease to one of my patients is low	Nurses	43	30.71	4.03	2.44–6.66	*P* < 0.0001
	Physicians	43	12.99			
Vaccines’ benefits are uncertain	Nurses	31	22.14	3.4	1.95–5.9	*P* < 0.0001
	Physicians	30	9.06			
Vaccines should be required to work as Health Care professionals	Nurses	90	64.29	0.52	0.29–0.92	*P* = 0.0238
	Physician	254	76.74			
I am afraid of side effects caused by vaccinations	Nurse	32	22.86	5.76	3.13–10.6	*P* < 0.0001
	Physicians	20	6.04			
I trust the information I received on vaccines	Nurses	85	60.71	0.28	0.15–0.51	*P* < 0.0001
	Physicians	281	84.89			
Becoming immune naturally by contracting a disease is better than becoming immune with vaccinations	Nurses	30	21.43	7.07	3.63–13.75	*P* < 0.0001
	Physicians	15	4.53			
Children should receive less vaccines in one visit	Nurses	47	33.57	6.44	3.78–10.96	*P* < 0.0001
	Physicians	31	9.37			
Children receive too many vaccinations	Nurses	21	15.00	4.85	2.37–9.91	*P* < 0.0001
	Physicians	14	4.23			
Overall, do you have doubts/worries regarding vaccines? 1 (a lot of doubts) to 5 (no doubts at all)	Nurses	76	54.29	0.19	0.11–0.33	*P* < 0.0001
	Physicians	289	87.31			

Employing the PACV Short Scale to evaluate vaccine hesitancy, 95.4% of physicians, 66.4% of nurses and 63.6% of other Health care professionals were ‘Not hesitant’ (score 0–4) (Table 8).

**Table 8:** Hesitancy level in Health Care professionals according to the PACV Short Scale *(not hesitant: 0–4 scores)*

PAVC Short Scale	Role			Total
	Others	Nurses	Physicians	
0	7	26	200	233
1		15	36	51
2	4	24	53	81
3	4	17	15	36
4	6	11	12	29
5	3	18	4	25
6	5	11	4	20
7	1	4	1	6
8	2	8	3	13
9	1	1	1	3
10		5	2	7
Total	33	140	331	504

The last question was ‘Would you like to express your opinion on this topic?’. The most mentioned issues were: the need for education on risks and benefits of vaccines because of its absence during training (24 on 101 answers) and the desire of being vaccinated for free at work (12 on 101 answers) without ‘any expenses, also time-wise, because vaccines are needed for the safety of patients’.

## Discussion

The data collected confirms that adherence of Italian health care professionals to influenza vaccination is far from recommended levels, as confirmed by other studies performed in Italy.

Analyzing our sample, in 2017–18 only 6 out of 10 physicians, 2 out of 10 nurses and less than 1 out of 10 of other health care professionals had vaccinated against influenza. At the same time, adherence to vaccination was lower in health care professionals with less than 40 years of age. Such data shows there was not enough focus on vaccinal prevention during their training.

Compared to physicians, nurses and other health care professionals were less trustworthy of the influenza vaccine, less aware of the possibility of contracting influenza and other vaccine-preventable diseases and transmitting them to their patients. Also they were unsatisfied of the information received on vaccinations, especially as far as influenza was concerned.

Such matter should worry professionals caring for high-risk patients, both in the pediatric and adult age, especially in emergency departments [23].

Studies performed during the 2009 H1N1 influenza pandemic showed that 50% of the health care professionals who contracted influenza had actually become infected while working in the hospital, contracting the virus either from patients or other health care professionals [24]. The highest rates of infection were noticed in health care professionals working in adult and pediatric emergency departments [19].

During the influenza season, and even more during epidemics, there is higher demand of medical care in hospitals [25]. Hospitals become overwhelmed with urgent and complex cases and, especially in pediatrics, also with not urgent patients. This happens either because families cannot access primary care [26], or because they have a broad concept of urgency [27, 28]. When more patients must be assisted, more health care professionals are needed. If health care professionals contract influenza themselves, the system will crumble. Therefore, it is important that health care professionals protect themselves through routine hygienical procedures as well as vaccines [29, 30].

The National Vaccine Prevention Plan (PNPV) 2017–19 highlighted that ‘every hospital should actively promote initiatives to increase adherence to vaccines in health care professionals and Health care students during the annual vaccine campaign held in Autumn’ [31]. At the same time, the Ministry of Health recommended to administer the influenza vaccine to all health care professionals ‘especially those working in departments at high risk of contracting and transmitting influenza, such as emergency departments and intensive care units, etc.’ [30].

The influenza vaccine is free for health care professionals because it is part of the Essential Levels of Care (LEA) [32]. Nevertheless, vaccine coverage is still low in physicians and, even more in nurses and other health care professionals [11, 12, 33–37]. Commonly, in a department nurses are more in number than physicians and considering also other health care professionals, it is clear that such low rates of vaccinations cannot be accepted and interventions should be implemented to increase vaccine coverage.

### What are the possible strategies?

To promote a higher adherence, a large quantity of tools have been employed, including memos, posters, fliers, text messages, emails to ‘gently push’ [38] health care professionals to vaccinate, together with educational activities on vaccines, open access to vaccine centers and vaccinations at the workplace [39, 40].

In the ‘IRCCS Ospedale San Martino di Genova’ the unit of Hygiene offered the influenza vaccine on the spot to health care professionals of departments at high-risk of infection, collecting their informed consent or dissent and thus increasing vaccine coverage in those departments compared to the others [12]. A similar initiative was carried out in the ‘IRCCS Ospedale Pediatrico Bambino Gesù of Rome’ during October–December 2017 where a communication campaign was held, promotion activities were organized and access to the influenza vaccination was facilitated: there a was statistically significant increase in vaccine coverage compared to the previous season [38].

In 2013 New York State obligated not vaccinated health care professionals to wear surgical masks when in patients’ areas. After this policy was implemented, more health care professionals accepted vaccinations and there was a reduction of health care professionals affected by respiratory diseases and laboratory confirmed influenza [41].

All these procedures are effective in increasing vaccine coverage in health care professionals but only compulsory vaccination allows to reach high levels of vaccinations. The American Academy of Pediatrics has no doubts. Compulsory vaccination for health care professionals is ethical, fair and needed to improve patients’ safety. It is a crucial step forward to reduce hospital-acquired influenza infections and optional vaccination is not sufficient to increase vaccine coverage [42].

Siemieniuk et al. [43] measured that compulsory vaccination determines a reduction of 93% (IC 95% 91–95%) of not vaccinated health care professionals, compared to the 74% reduction reached with vaccine or mask, to the 28–41% reached with dissent modules, audits and feedback, facilitated access to vaccination, experienced colleagues promoting vaccinations (peer vaccinator), whereas education was associated with the lowest reduction, 11% (IC 95% 7–16%).

It is interesting that 66.2% of physicians and 42.9% of nurses who participated in our survey thought that the influenza vaccine should be required to work as health care professionals. Even more professionals agreed on compulsory vaccinations as a whole: 76.8% of physicians and 64.3% of nurses.

Is compulsory vaccination a possible strategy in Italy? In June 2018 the region Puglia approved a law [44] imposing to perform the vaccinations included in the PNPV 2017–19 to all workers at risk. Emilia Romagna and Marche, considering the law ‘D.lgs. 9 aprile 2008, n. 81’ [45] established that vaccinations are required to work in emergency departments, pediatrics and neonatology.

Although limited, our data highlights the need to promote vaccine education in hospitals and during training to allow students of medical schools, residency programs and other health care degrees, to be properly informed and receptive to accept and promote themselves recommended vaccinations.

Our study has some limits. The research was conducted with an online survey open to all health care professionals, also those who were not members of SIMEUP. Hence, the results obtained should not be considered as representative of the opinion of all SIMEUP members. Moreover, considering the fluctuation of vaccination rates observed in Italy in recent times, our sample can hardly represent the entire population. Nevertheless, the geographical heterogeneity of the participants should guarantee a certain level of consistency about the real situation of health care professionals in Italy.

## Conclusions

In the document ‘*La Carta di Pisa delle vaccinazioni negli operatori sanitari*’ [46] endorsed in March, 2017 by several researchers and seven scientific societies, including SIP, the ‘absolute importance of vaccinations in health care professionals to achieve vaccine-preventable diseases control’ was reiterated.

SIP and SIMEUP invite their members to vaccinate as soon as possible against influenza, to become a vaccine champion and to advocate for all preventive measures required by law to be implemented in emergency departments and other high-risk sectors.

‘High vaccine coverage of health care professionals should be embedded in the safe hospital paradigm and/or should become a goal for hospitals’ directors [47].

## Abbreviations

O.R.: Odds Ratio
PACV: Parent Attitudes about Childhood Vaccines
PNPV: National Vaccine Prevention Plan
S.D.: Standard Deviation
SIMEUP: Italian Society of Pediatric Emergency Medicine
SIP: Italian Society of Pediatrics
